# Light-emitting diode stimulates radiodermatitis recovery

**DOI:** 10.1590/ACB360301

**Published:** 2021-02-26

**Authors:** Cristina Pires Camargo, Heloisa Andrade Carvalho, Feres Camargo Maluf, Alexandre Agostinho da Cruz Sousa, Paulo Otavio Maluf Perin, Marcela Maluf Perin, Julio Morais-Besteiro, Rolf Gemperli

**Affiliations:** 1 MD, PhD. Universidade de São Paulo – Medical School – Laboratory of Microsurgery and Plastic Surgery – São Paulo (SP), Brazil.; 2 MD, PhD. Universidade de São Paulo – Medical School – Department of Radiology and Oncology – São Paulo (SP), Brazil.; 3 Graduate student. Centro Universitário de Saúde ABC – School of Medicine – Santo André (SP), Brazil.; 4 Graduate student. Universidade de São Paulo – Medical School – Laboratory of Microsurgery and Plastic Surgery – SãoPaulo (SP), Brazil.; 5 Graduate student. Centro Universitário Lusíadas – School of Medicine – Santos (SP), Brazil.; 6 PhD. Universidade de São Paulo – Medical School – Laboratory of Microsurgery and Plastic Surgery - São Paulo (SP), Brazil.

**Keywords:** Radiotherapy, Lasers, Wound Healing, Radiodermatitis, Rats

## Abstract

**Purpose:**

To evaluate the effect of light-emitting diode (LED) in an experimental model of radiodermatitis.

**Methods:**

Ten male Wistar rats weighing 200–250 g were analyzed. Radiation was delivered in a single dose (20 Gy with Strontium-90 dermatological plaques), two areas per animal. After 15 days, they were divided into two groups: control group (n = 5) and LED group (n = 5), which was treated during 21 days later (LED 660 nm, 10 min in alternate days). The endpoints were radiodermatitis scale, histological analysis HE, Picrius Sirius and the gene expression of interleukin-10 (IL-10) and matrix metalloproteinase-9 (MMP-9).

**Results:**

The LED group showed a higher number of dermal appendages (p = 0.04) and angiogenesis(p = 0.007), a tendency towards higher IL-10 (p = 0.06) and an increase in MMP-9 (p = 0.004) when compared to the control group.

**Conclusions:**

This study suggested that the use of LED for radiodermatitis increased skin regeneration.

## Introduction

Radiotherapy is one of the cornerstones of cancer treatment, along with surgery and chemotherapy. It is estimated that more than 50% of cancer patients have received irradiation in some part of their treatment, either with curative or palliative intent^1^–^3^. Radiation is mostly delivered by external beams that enter and exit the body through the skin, thus, nearly 85–90% of patients submitted to radiotherapy have some degree of radiodermatitis^4^.

The ionizing effects of radiotherapy cause the breakdown of DNA of cancerous and normal cells. These injuries make cell repair impossible and consequently cause cell death and loss of skin continuity^1^,^2^.

Radiodermatitis depends on the total radiation dose and fractionation, comorbidities, smoking, associated treatments (use of chemotherapy) and patient sensitivity, among others. Signs and symptoms range from mild local burning sensitivity to skin ulcerations in the irradiated area. Radiodermatitis can also be classified according to the time of onset for the appearance of symptoms in acute (in the first 90 days after irradiation) or chronic (months and years after irradiation)^1^,^5^,^6^.

In clinical practice, patients who have these most severe complications share a low quality of life due to the pain caused by irradiation lesions, as well as the increase of medical visits and local care.

Some therapeutic alternatives are used to prevent and mitigate the symptoms of radiodermatitis, such as topic (moisturizing creams, lotions, topical corticosteroids) and systemic treatments (corticosteroids, antioxidants)^6^,^7^.

However, according to Chan *et al*.^7^ systematic review, there is no consensus on which of these therapeutic alternatives would be more effective in the treatment of radiodermatitis*7*.

One of the medical strategies is the use of low-level laser therapy (LLLT). This treatment uses nonionizing forms of lasers that triggers acceptors (intracellular structures receptive to photons). In several studies, PBM therapy showed significant efficacy in preventing radiodermatitis for breast cancer. But there is a lack of data to show the effect of LLLT to treat radiodermatitis^8^.

The hypothesis is that the LED treatment could decrease inflammatory cytokines, increase neo-vascularization in the lesion. To test this hypothesis, the clinical wound healing process, histologic structures (dermic appendages, angiogenesis) and biomarkers (interleukin-10 [IL-10] and matrix metalloproteinase-9 [MMP-9]) were analyzed.

Due to this lack of evidence and based on clinical experience, this study was performed to evaluate the effect of LED in the treatment of acute radiodermatitis in rats submitted to ionizing radiation.

## Methods

This study followed the national standards of good practices in animal care according to the CONCEA guidelines and was approved by CEUA-FMUSP under registration 1060/2018.

Ten male Wistar rats, age 8 weeks, weighing 200–250 g were analyzed. These animals were submitted to a single dose of radiotherapy session. After fifteen days post-radiotherapy, the animals were divided into two groups.

### Irradiation of the dorsal region

All animals were anesthetized by an intraperitoneal injection of the association of ketamine hydrochloride (Ketamin, Cristalia, Brazil) 100 mg/kg and xylazine hydrochloride (Rompun, Bayer, Brazil), 5 mg/kg. They were placed in ventral decubitus and an area of 10 × 6 cm in the back of the animal was trichotomized.

Two strontium plaques were applied per animal (dorsal region to reduce sample size). The areas of radiation were at least 3 cm apart to avoid crossover effect.

Radiation was delivered with two strontium-90 (90Sr) dermatological plaques that emitted beta radiation (model SIQ21, with reference dose rate = 0.051 Gy/s and Model SIQ18 with reference dose rate = 0.048 Gy/s, Amersham International plc). Two areas of 2 × 2 and 2 × 1 cm in the dorsum of each animal were exposed to a single dose of 20 Gy^8^–^10^.

After the radiotherapy session, the animals were kept in vivarium for 15 days until the radiodermatitis lesions onset.

After this period, they were divided into two groups:

- Control group: n = 5, no further treatment;
- Intervention group LED: n = 5, exposure to LED (wavelength 660 nm), one session lasting 10 min, on alternate days, for the following 21 days.

### Light-emitting diode parameters

In the intervention group, the animals were kept in a plastic cage (8 × 10 cm). On the top of the cage, a LED device was placed.

The LED equipment parameters were wavelength 660 nm, irradiance 1050 W/cm^2^, energy density 5 J/cm^2^.

The treatment regimen: three times in the week, one daily exposition, 10 min of exposure for three weeks (seven treatment sessions).

### Macroscopic analysis of radiodermatitis

The radiodermatitis lesions were analyzed in two periods: pre- (15^th^ post-irradiation day) and post-treatment (21^st^ day of treatment). After the 15^th^ post-irradiation day and in the 21^st^ day of treatment with LED, the dorsal region of the animals was photographed (Canon EOS Rebel T7 DSLR, 24.1 MP, Canon, USA). Two independent investigators analyzed the dorsal skin area before 15 post-radiotherapy session and every week during the three weeks of treatment and reactions were classified using the radiation therapy oncologic group scale (RTOG)^9^ (Table 1).

**Table 1:** Radiation therapy oncologic group scale (RTOG).

Grade	RTOG scale
Grade 1	Normal appearance
Grade 1.5	Minimal erythema
Grade 2	Moderate erythema
Grade 2.5	Erythema associated with dry flaking
Grade 3	Erythema associated with confluent dry desquamation
Grade 3.5	Confluent dry flaking, crusts
Grade 4	Moist flaking, moderate scabs
Grade 4.5	Wet peeling, small ulcers
Grade 5	Large ulcers
Grade 5.5	Necrosis

### Microscopic analysis

At the end of 21 days after the groups’ assignment, the animals were euthanized by an intraperitoneal injection of ketamine hydrochloride (Ketamin, Cristalia, Brazil) 150 mg/kg and xylazine hydrochloride (Rompun, Bayer, Brazil), 10 mg/kg.

Two samples from each lesion were collected. One was prepared for histological analysis and the other was frozen with Nitrogen -80 °C and was performed to analyze IL-10 and MMP-9.

### Histological analysis

One sample was fixed in 4% formalin for 24 h, embedded in paraffin for hematoxylin-eosin (HE) and Picrius Sirius staining.

The vascular density (angiogenesis), dermic appendages (number of hair follicle) were quantified by HE staining under optical microscopy (Nikon eclipse E600, Japan) magnification (× 20, × 40). And by Picrius Sirius staining, the collagen fibers distribution and density (graphic distribution structure) were analyzed. All histological structures mentioned above were analyzed and quantified in 10 fields per slide.

### Analysis of gene expression

#### Total RNA extraction

The samples of fleshy panicle were macerated using the Tissue Lyser LT apparatus (Qiagen, Germantown, USA), 1.0 mL of Trizol (Invitrogen-Life Technologies, Carlsbad, USA) and stainless-steel beads were added to the microcentrifuge tubes. Fragmentation was carried out for 6 min at 50 Hz.

After removing the beads, 0.2 mL of chloroform (Merck, USA) was added. The samples were centrifuged for 15 min at 12,000 rpm at 4 °C. After centrifugation, the aqueous phase was transferred to a new microcentrifuge tube and 0.5 mL of cold isopropyl alcohol (Merck, USA) was added to precipitate the RNA. The samples were incubated at room temperature for 10 min and then centrifuged at 12,000 rpm for 10 min at 4 °C. The supernatant was discarded and the precipitate, containing RNA, was washed with 1.0 mL of 75% ethanol. It was centrifuged for 5 min at 10,000 rpm at 4° C. The flask containing RNA was resuspended in 50 to 100μL of sterile ultrapure water free of DNase/RNase (Invitrogen-Life Technologies, Carlsbad, USA).

The concentration of the extracted RNAs was determined on a NanoDrop ND-1000 spectrophotometer (NanoDrop Technologies, Inc., Wilmington, USA). The degree of purity was evaluated by the ratio 260/280 nm, using only RNAs which ratio was ≥ 1.8. For the analysis of the integrity of the RNAs, electrophoresis in agarose gel was performed to check the 28S and 18S bands. The extracted RNAs were stored at -80 °C until use. The degree of purity of the RNA was confirmed with the average ratio 3 1.9.

#### Synthesis of cDNA

For the synthesis of cDNA from the total RNA, the high-capacity RNA-to-cDNA kit (Applied Biosystems) was used in a GeneAmp 2400 thermocycler (Applied Biosystems). In final volume of 20 μL: 1.0 μL of enzyme mix; 10.0 μL of RT buffer; qsp 20 μL of sterile ultrapure water free of DNase/RNase and total RNA (500 ng). For the reaction and stopping the reaction, the tubes were incubated at 37 °C for 60 min and at 95 °C for 5 min, respectively. The cDNA samples were stored at -20 °C until use.

#### Real-time polymerase chain reaction (qRT-PCR)

The analysis of gene expression of the mRNA levels of interest was performed by qRT-PCR in the StepOnePlus thermocycler (Applied Biosystems) with the TaqMan Gene Expression Assays system (Applied Biosystems).

The probes and primers for the C5AR1 (Rn02134203), ICAM 1 (Rn 00564227), iNOS (Rn 00561646), VEGF (Rn 01511602) and for the endogenous control ACTB (Rn 00667869) were acquired from the company list of inventoried assays Applied Biosystems.

Real-time polymerase chain reaction was performed in duplicate for each sample using: 10.0 μL TaqMan Universal Master Mix II 2X, 1 μL TaqMan Gene Expression Assay 20 × and 4 μL of diluted cDNA (dilution 1:5) in a final volume of 20 μL, in 96-well plates covered with optical sealant.

The reaction conditions were as follows: temperature of 50 °C for 2 min, 95 °C for 10 min, followed by 40 cycles at 95 °C for 15 s and 60 °C for 1 min.

To calculate the expression level of each target gene, the GenEx Standard 6.1 software (MultiD Analyzes AB) was used, which uses the 2-delta delta Ct method for relative quantification, where Ct (threshold cycle) is the qRT-PCR, in which the amplification reaches the logarithmic phase, where delta Ct is the difference in expression between the target gene and endogenous control of a given sample and 2 ^ delta delta Ct values corresponds to the difference between the 2 ^ delta delta Ct of the sample and the 2 ^ delta delta Ct of control.

#### Statistical analysis

Because of the small sample size, the two groups variables were compared using the Wilcoxon rank sum test (non-normal distribution), considering an alpha p of 0.05 and 80% power. Statistical software STATA version 14 (StataCorp, 2015, Stata Statistical Software: Release 14. College Station, TX: StataCorp LP) was used for calculation.

## Results

There were no complications or drop outs in this study.

### Macroscopic analysis of radiodermatitis

The radiodermatitis lesion was analyzed in two periods. In the first, immediately before the LED treatment (after 15 days of radiation exposure), all animals developed grade 5 radiodermatitis.

**Figure 1:**
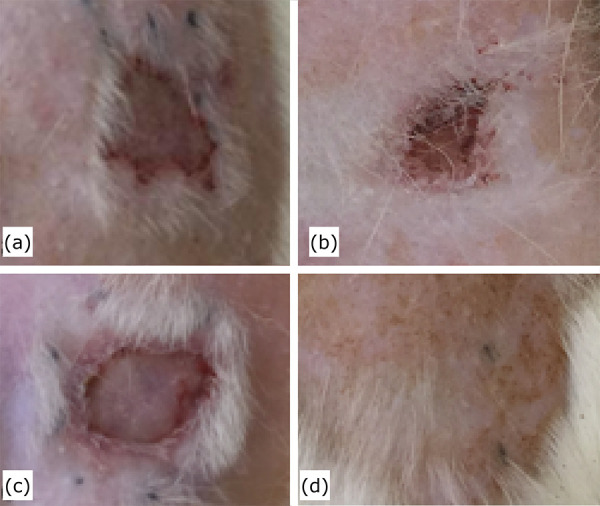
Macroscopic aspect of radiodermatitis. (a) Control – pretreatment (RTOG scale score 5). (b) control – post-treatment (RTOG scale score 4.5). (**c**) LED – pretreatment (RTOG scale score 5), (**d**) LED = light-post-treatment (RTOG scale score 1)

The second follow-up was after 21-days of treatment (control and intervention). The lesions treated with LED showed grade 1 radiodermatitis (RTOG scale) and the control group still presented radiodermatitis lesions classified by the RTOG scale as grade 4.5 (Fig. 1).

### Microscopic analysis

**Figure 2:**
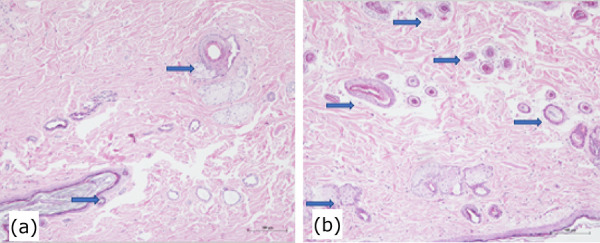
Hematoxylin-eosin staining. (**a**) LED group, (**b**) control group. The LED treatment showed a high number of arterioles (angiogenesis) and dermal appendages when comparing to the control group.

In HE preparation, the study showed an increase of dermal appendages (p = 0.04) and an increase in neoangiogenesis (p = 0.007) in the group treated with LED when compared with the control group (Table 2) (Fig. 2).

**Table 2:** Description of histological structures (dermal appendages and neoangiogenesis) in HE.

Group	Dermic appendages (mean ± SD, units)	Neoangiogenesis (mean ± SD, units)
Control	8.4 ± 2.0	3.25 ± 1.0
LED	11.5 ± 3.1	27.3 ± 13.4

LED = Light-Emitting Diode; SD = Standard Deviation

**Figure 3:**
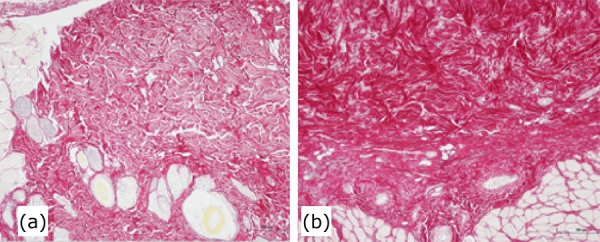
Picrius Sirius staining. (**a**) LED group, (**b**) Control group. The LED treatment group treated with LED showed an increase in young type collagen (fine fibers) compared to the control group.

In the analysis of Picrius Sirius red, the group treated with LED showed an increase in young type collagen (fine fibers) compared to the control group (Fig. 3).

### Analysis of gene expression

This study showed a tendency to increase IL-10 gene expression level in the group treated with LED when compared to the control group (p = 0.06). The analysis of MMP9 showed an increase of MMP9 gene expression in the group treated with LED when compared to the control group (p = 0.005) (Table 3).

**Table 3:** Analysis of gene expression of IL-10 and MMP9 in the group treated with LED and control group.

Group	IL-10 (mean ± SD, units)	MMP9 (mean ± SD, units)
Control	1.76 ± 1.0	5.59 ± 3.6
LED	3.11 ± 1.5	116.13 ± 52.8

LED = Light-Emitting Diode; SD = Standard Deviation

## Discussion

The use of low voltage laser (low-power LED), also known as photobiomodulation, has become an attractive therapeutic option for tissue recovery, because of its non-invasive nature^1^–^3^. This study analyzed the effect of photobiomodulation in experimental radiation induced dermatitis on the dorsal skin of rats.

The results showed that photobiomodulation (LED – 660 nm wavelength) promoted a faster healing of the radiation-induced skin lesions.

The macroscopic analysis of the radiodermatitis lesions showed a better tissue regeneration in the LED group when compared to the control. The LED treated group started treatment with radiodermatitis grades ranging from 4 to 5. After the LED sessions ended, these lesions were classified as grade 1. As for the control group, there was no change in the degree of radiodermatitis (grade 4.5).

Furthermore, a stimulation in the formation of dermal appendages and neoangiogenesis were verified in those lesions. According to these findings, it is possible to infer that LED treatment increased the ability of tissue regeneration in radiodermatitis lesions in rats when compared to the control group.

There are few studies that analyzed the action of LED on radiodermatitis. Nishioka *et al*.^12^ analyzed the effect of LED on the viability of random flaps in healthy rats compared to a sham group and groups treated with two different energies of LLLT. They found that LED was more effective in increasing the number of mast cells and blood vessels in the transition line of random skin flaps. The increase of arterioles (neoangiogenesis) is crucial for the wound healing process resolution. In the literature data, radiodermatitis lesions showed a great number of fibrotic fibers. The increase of angiogenesis balances the macrophage phenotype 1 and 2 to decrease the fibrosis process for neoangiogenesis to promote wound healing^11^–^13^.

Peralta-Mamani *et al*.^14^ demonstrated that the use of LLLT in patients with mucositis resulting from radiotherapy of head and neck cancer showed a decrease in pain levels and increased mucosal regeneration.

The hypothesis of the present study is based on the effect of LED as a factor that would stimulate the release of cellular and humoral anti-inflammatory factors causing regeneration of radiodermatitis.

In this sense, the analysis of the collagen fibers by the Picrius Sirius Red staining method showed an increase in fine, young collagen fibers in the group of animals treated by LED. Light-emitting diode photobiomodulation increased tissue regeneration, probably by stimulating fibroblasts to secrete fine collagen fibers at the expense of the release of myofibroblasts that could cause contraction and fibrosis of the lesion by radiodermatitis.

In fact, several studies have demonstrated the anti-fibrotic action of low voltage laser^14^–^16^. Chang *et al*.^16^ used low voltage lasers (463 nm wavelength) on the skin of young and elderly mice. This study showed a lower gene expression of proteolytic biomarkers (TGF-beta, collagen types I and II) in the low-voltage laser group. The formation of new collagen corroborates with the fact that there is an increase in the proliferation of fibroblasts in the expense of the production of myofibroblasts in the healthy skin of young and elderly animals.

Similarly, the present study showed that the use of low voltage laser for the treatment of radiodermatitis would stimulate the proliferation of fibroblasts and formation of new collagen that would cause less fibrosis in the regeneration of radiodermatitis, as observed in the group treated with LED.

As for the analysis of gene expression of healing biomarkers, photobiomodulation showed a tendency to increase the levels of IL-10 gene expression. This finding was similar to the study by Rambo *et al*.^15^. These authors used low-intensity laser on healthy skin and analyzed the gene expression of inflammatory and anti-inflammatory factors. Rambo *et al*.^15^ demonstrated that the use of low-level laser increased the IL-10 gene expression. The increase in IL-10 can be explained by the stimulation of acceptors (chromophores) located in the mitochondrial membrane. The activation of this acceptor increases the production of ATP, proteins and cell proliferation^13^–^16^.

Interestingly, the present study demonstrated an increase in MMP9 in the LED treated group when compared to the control group. The literature regarding this finding is conflicting. Some authors have demonstrated that the use of low voltage laser (430 to 630 nm) decreases, in a short term, the gene expression of metalloproteinases^15^,^16^. Others demonstrated an increase in MMP9 gene expression in second degree burns in rats on the 18^th^ day after the injury^17^. The difference between the results of MMP9 gene expression in the studies can be explained by the use of different laser sources, energy intensity, wavelength, as well as the data analysis timing (short or long term). The findings are compatible with the study by Maligieri *et al*.^18^ that used different energy densities for treatment. In addition, there are indications that MMP9 may play a role in physiological and pathological tissue remodeling of central nervous system through a new regulatory pathway of TGF-β1 in brain astrocytes^18^.

The laser source used may be considered a limitation of this study. The 660 nm intensity stimulates tissue regeneration, however, the association of two wavelengths (660 and 830 nm) that act synergistically in tissue regeneration may be more efficacious^19^–^21^. Despite that, the findings demonstrated a better recovery capacity of the lesions treated with the 660 nm LED.

Still, the study of other biomarkers would be interesting, such as TNF-alpha, and VEGF-alpha, in addition to the analysis of genes related to healing and apoptosis.

## Conclusions

The use of LED (660 nm) increased the regeneration process of radiodermatitis lesions in rats when compared to the control group. Light-emitting diode treatment increased the proliferation of dermal appendages and neoangiogenesis, as well as the proliferation genes expression IL-10 and MMP9.
